# Society Meetings

**Published:** 1904-02

**Authors:** 


### SOCIETY MEETINGS.

The Southern Surgical and Gynecological Association at its annual meeting, held in Atlanta, December 15-17, 1903, elected the following named officers for the ensuing year: president, Floyd W. McRae, Atlanta; first vice-president, George S. Brown, Birmingham; second vice-president, J. Shelton Horsley, Richmond; treasurer, Charles M. Rosser, Dallas, Tex; secretary, William D. Haggard, Nashville. Dr. Richard Douglas, of Nashville, presented a memorial of Dr. William E. B. Davis, which was an eloquent and appropriate tribute to the distinguished founder of the association. The next annual meeting will be held at Birmingham, December 13-15, 1904, and Dr. John D. S. Davis will act as chairman of the committee of arrangements.

The first French congress of climatotherapy and hygiene of towns will be held at Nice, from April 4 to 9, 1904, under the presidency of Professor Chantemesse. The following named five subjects will be discussed: (1) the climate of the French Mediterranean coast; (2) adaptation of the individual to climate; (3) influence of the French Mediterranean coast climate on tuberculosis and tubercular patients; (4) the influence of climate of the French Mediterranean coast on rheumatism and on those subject to rheumatism; (5) disinfection of towns. Dr. Hérard de Besse is secretary general of the congress, Beaulieu-sur-Mer.

The Mississippi Valley Medical Association will hold its thirtieth annual meeting at Cincinnati, O., October 11, 12, 13, 1904. Dr. B. Merrill Ricketts has been elected chairman of the committee of arrangements. The list of officers was published in the issue of the Journal for November, 1903.

The American Electrotherapeutic Association will hold its fourteenth annual meeting at Saint Louis, September 13-16, 1904, under the presidency of Dr. A. D. Rockwell, of New York. Dr. Ernest Wende, of Buffalo, is a member of the executive council. A booklet has been issued giving a historical sketch of the association, lists of officers, committees and members, together with other information. Dr. Charles Edward Skinner, of New Haven, is the secretary.

The seventh international congress of otology will be held at Bordeaux, France, August 1-4, 1904, under the patronage of the Minister of Public Instruction. The official languages of the congress will be French, English, German, and Italian. A museum devoted to the exhibition of surgical instruments, anatomical and pathological preparations concerning the ear, nasal fossæ, and nasal pharynx will be conducted during the congress.

The discussion of the following subjects will constitute the order of the day:

1. Choice of a simple and practical acoumetric formula—leaders: Mm. Politzer, Gradenigo, Delsaux.

2. Diagnosis and treatment of labyrinthine suppuration—leaders: Drs. Brieger, Von Stein, Dundas, Grant.

3. Technique and care of otogenous cerebral abscess—leaders: Drs. Knapp, Schmiegelow, and Botey.

The Prize Lenval, granted through the generosity of Baron Lenval, will be awarded at the congress for the most practical treatment of auditory affections during the past two congresses. One of the jurors to award this prize is Dr. D. B. St. John Roosa, of New York. Dr. Lermoyes, Rue de la Boltie 20, Paris, is secretary general of the congress.

---

THE Buffalo Academy of Medicine held meetings during the month of January, 1904, as follows:

*Section on Ophthalmology, Otology, Laryngology and Rhinology.*—The postponed meeting of this section was held at the University of Buffalo, on Monday afternoon, January 4, at 5 o'clock. Program: Clinical anatomy of the middle ear, illustrated by stereopticon, Professor B. A. Randall, president of the American Otological Society, and professor of diseases of the ear, at the University of Pennsylvania, Philadelphia.

*Section on Surgery.*—Tuesday evening, January 5. Program: Exploratory operations, their justifiableness, Eugene A. Smith; Should the surgeon and the radiographist work together? Illustrated by stereopticon, A. W. Bayliss.

*Section on Medicine.*—Tuesday evening, January 12. Program: Are we as physicians carriers of contagion? Lucien Howe; discussion by H. G. Matzinger, Herbert U. Williams, and Thos. B. Carpenter.

*Section on Pathology.*—Tuesday evening, January 19. Program: The practical issues in studying the nature of mental diseases, Adolph Meyer; discussion was opened by Drs. Putnam, Krauss, and Hurd.

---

THE American Medico-Psychological Association will hold its sixtieth annual meeting, beginning Monday, May 30, and ending Friday, June 9, 1904, at the Planters Hotel, Saint Louis,

Mo. Titles of papers should be sent to the secretary, Dr. C. B. Burr, Flint, Mich.

---

THE American Public Health Association, at its recent meeting in Washington, D. C., elected the following named officers: president, Carlos J. Findlay, Havana, Cuba; first vice-president, J. R. Monjaras, Mexico; second vice-president, William C. Woodward, Washington, D. C.; secretary, Charles O. Probst, Columbus; treasurer, Frank W. Wright, New Haven.

---

THE Western Surgical and Gynecological Association, at its recent annual meeting at Denver, elected the following named officers for the ensuing year: president, Charles H. Mayo, Rochester, Minn; first vice-president, H. D. Niles, Salt Lake City; second vice-president, L. L. McArthur, Chicago; secretary-treasurer, B. B. Davis, Omaha.

